# Supplementary material for: An indirect method to monitor the fraction of people ever infected with COVID-19: An application to the United States
Source: PLoS One. 2021 Jan 28;16(1):e0245845. doi: 10.1371/journal.pone.0245845 (PMC7842943; doi:10.1371/journal.pone.0245845)
Supplement: S1 File — (PDF) [file pone.0245845.s001.pdf]

An indirect method to monitor the fraction of people ever infected with COVID-19: An application to the United States

Miguel Sánchez-Romero, Vanessa di Lego, Alexia Prskawetz, Bernardo L. Queiroz

S1 Material and methods

S1.1 Model

To predict the evolution of the number of deaths caused by the epidemic we extend a standard epidemiological age-structured SEIR (susceptible-exposed-infected-removed) model by (a) accounting for the age-specific mortality rates and (b) explicitly modeling the mortality rate of the COVID-19 epidemics. To account for the differential effect of mortality by age, each state is comprised of 95 (Ω) age-groups. We distinguish vectors and matrices from scalars by using bold letters. The dynamics of our age-structured SEIR model are as follows:

$$\begin{aligned}
\dot{\mathbf{S}}_t &= -\beta_t \mathbf{S}_t - \mathbf{M}\cdot\mathbf{S}_t \\
\dot{\mathbf{E}}_t &= \beta_t \mathbf{S}_t - \delta\mathbf{E}_t - \mathbf{M}\cdot\mathbf{E}_t \\
\dot{\mathbf{I}}_t &= \delta\mathbf{E}_t - \nu\mathbf{I}_t - \mathbf{M}\cdot\mathbf{I}_t \\
\dot{\mathbf{R}}_t &= \nu(\mathbf{1}-\mathbf{F})\mathbf{I}_t - \mathbf{M}\cdot\mathbf{R}_t \\
\dot{\mathbf{D}}^c_t &= \nu\mathbf{F}\cdot\mathbf{I}_t
\end{aligned}$$

where $\beta_t = \beta[\sum_{x=0}^{\Omega}\mathbf{I}_t(x)/\sum_{x=0}^{\Omega}(\mathbf{S}_t(x)+\mathbf{E}_t(x)+\mathbf{I}_t(x)+\mathbf{R}_t(x))]$ is the infection rate and β is the effective transmission rate, $\mathbf{S}_t$ denotes the vector of susceptible individuals. $\{\mathbf{E}_t, \mathbf{I}_t\}$ denote the vector of exposed and infectious individuals, respectively. $\mathbf{R}_t$ is the vector of recovered individuals and $\mathbf{D}^c_t$ is the vector of deaths from the COVID-19 outbreak. $\mathbf{M}$ and $\mathbf{F}$ are, respectively, diagonal matrices with age-specific death rates not caused by COVID-19 and the estimated age-specific infection fatality rates for the COVID-19. The infection fatality rate (hereinafter IFR) is defined as the ratio between the total number of deaths from COVID-19 and the total number of infected individuals from COVID-19. Note that unlike IFR, the case fatality rate (hereinafter CFR) is the ratio between the total number of deaths from COVID-19 and the total detected infected individuals. $\mathbf{1}$ denotes the identity matrix. The set of parameters $\{\beta, \delta, \nu\}$ denotes the effective transmission rate of the disease, the inverse of the incubation period, and the removal rate.

The dynamics are presented in the flow diagram Figure S1. In each time step (day) a susceptible individual may become exposed with probability β_t. An exposed individual spends an average period of δ^{-1} days in incubation until becoming infectious. After an average period of ν^{-1} days, infected individuals can either recover with probability $1-\mathbf{F}$ or die due to COVID-19 with probability $\mathbf{F}$.

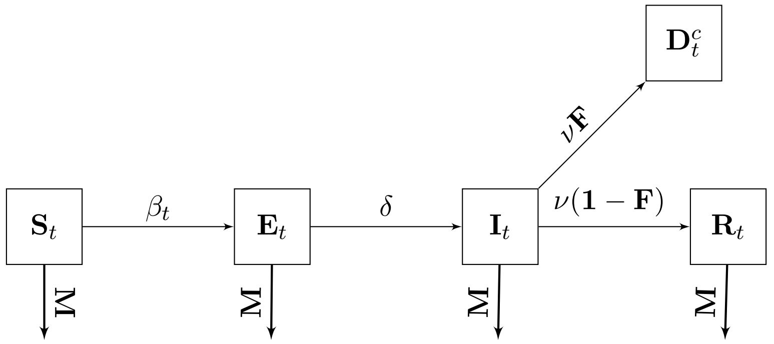

Figure S1: Schematic diagram of the SEIR model with deaths from COVID-19.
Notes: Bold letters $\mathbf{S}_t, \mathbf{E}_t, \mathbf{I}_t, \mathbf{R}_t$ denote, respectively, the vector of susceptible, exposed, infected, and recovered individuals at each age, while $\mathbf{D}_t^c$ is the vector of deaths at each age from the COVID-19 outbreak. The terms $\mathbf{M}$ and $\mathbf{F}$ are, respectively, diagonal matrices with age-specific death rates not caused by COVID-19 and the estimated age-specific infection fatality rates for the COVID-19. Parameters β_t, δ, ν represents, respectively, the infection rate, the inverse of the incubation period, and the removal rate.

S1.2 Data and calibration

The COVID-19 outbreak is characterized by a large uncertainty on the number of people infected. The number of deaths from COVID-19 seems more reliable despite the fact that it can also be subject to under-reporting [12] as well as to over-reporting, due to competing causes of death. Under-reporting occurs because there is no common agreement across countries on how COVID-19 deaths should be counted. Indeed, many countries are only counting as COVID-19 deaths those individuals who were tested positive, despite the fact that many people who died with COVID-19 symptoms were not tested. To avoid under- and over-reporting, we take into account in the model not only deaths from COVID-19, but also all the expected deaths that would have occur without the pandemic by introducing the matrix of age-specific death rates $\mathbf{M}$.

Death rates. The vector of age-specific death rates not caused by COVID-19, $\mathbf{m}$, for each U.S. state is taken from [21]. Thus, we have that $\mathbf{M} = \text{diag}(\mathbf{m})$.

Statistics on deaths by COVID-19 have shown a sizable age gradient [8], which is quite similar across countries as shown by [9]. For this reason, it is important to consider in the model that COVID-19 fatality rates are increasing with age. To account for the age pattern, we regressed through an OLS the function: $\log \text{cfr}(x) = \gamma_0 + \gamma_1 x + \gamma_2 x^2 + v$, where v is the error term, to the log of COVID-19 age-specific fatality rates for ages older than 30 years from [19].[1] We denote the vector of regressed case fatality rates as $\mathbf{m}^c$. The dotted blue line in Figure S2 shows the fit of our regression function to the data (red squares) where $\gamma_0 = -10.5063$, $\gamma_1 = 0.1310$, and $\gamma_2 = -0.0003$. To control in our estimates for the unknown number of underreported infected cases, we introduce in the calibration an adjustment factor $\varepsilon \in (0, 1)$

[1] Note that we do not fit the fatality rate data below age 30, given that the share of infected and asymptomatic individuals below age 30 is likely to be underreported.

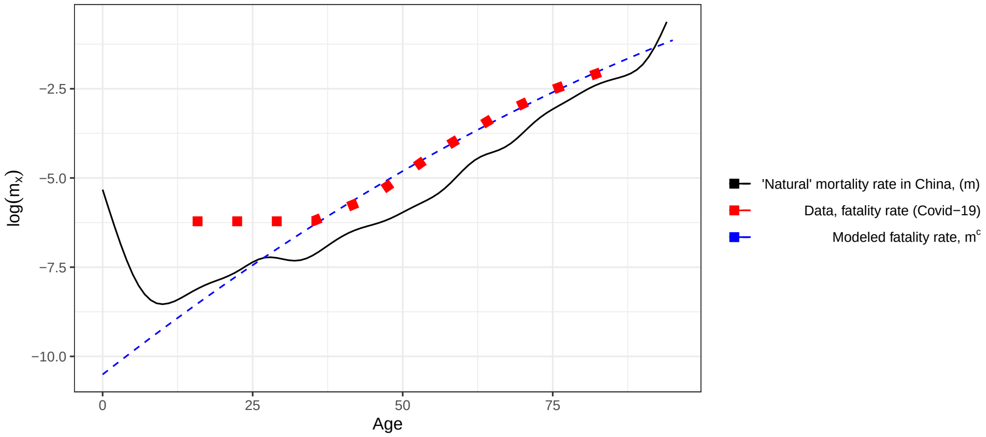

Figure S2: Fitted fatality rate for COVID-19. Source: "Natural" age-specific mortality rates (black solid line) were calculated using data from the UN Population Division. Age-specific data on COVID-19 case fatality rates is taken from [19].

that allows us to compute the infection fatality rate. Note that the number of underrerported infected cases includes, although not exclusively, asymptomatic individuals. Thus, the age-specific infection fatality rate implemented in the SEIR model is $\mathbf{F} = \mathrm{diag}(\varepsilon \cdot \mathbf{m}^c)$.

Population data. We took data by single year of age for each U.S. state using the July 2019 version from the Census Bureau.[2] In order to have the population size by single years of age from age 85 until age 94+, we ungrouped for each state the age group 85+ by using the person-years lived column from the state life tables calculated by [21].

Bayesian melding. Our strategy is to fit our SEIR model against the total number of deaths. Since the SEIR model implemented is deterministic and the data on deaths is likely incomplete, we implement the Bayesian melding method [15], which provides an inferential framework that takes into account both model's inputs and outputs. The basic purpose with the Bayesian melding method is to derive the distribution of the set of parameters that best replicate the observed evolution of deaths by using the information from the model and the data. The Bayesian melding has already been applied in epidemiology to study the HIV/AIDS prevalence [1; 6; 3; 4; 2]. Indeed, the UNAIDS uses this method for the official estimates of HIV/AIDS epidemic.

Let $\Theta \subseteq \mathbb{R}^4$ be our four dimensional set of parameters –inputs– to be estimated in our SEIR model. For simplicity, we denote by $M(\Theta)$ the application of our SEIR model given the inputs. Let a realization of Θ be $\theta = (\beta, \delta, \nu, \varepsilon)$. Each parameter is considered a random variable with a joint prior distribution $q_1(\Theta)$. We assume independent uniform priors for the distribution on the inputs.

$$q_1(\Theta) = \mathcal{U}([0.02,\, 2] \times [0.066,\, 2] \times [0.02,\, 2] \times [0,\, 1]). \qquad (1)$$

[2]We downloaded the table `Single Years of Age and Sex Population Estimates: April 1, 2010 to July 1, 2019 - CIVILIAN (SC-EST2019-AGESEX-CIV)`.

The domains of parameters β, δ, ν, and ε are set so as to consider any potential value from the existing literature [e.g. 16].

Let Φ be the set of outputs of our SEIR model. Given that $M(\Theta) = \Phi$, the outputs Φ are also a random variable with a joint prior distribution $q_2(\Phi)$. Let a realization of Φ be $\phi = \{e_s\}_{s=1}^{T}$, where the output e_s is the difference between the model's total number of deaths ($\mathbf{D}_s^c$) and the total observed deaths from COVID-19 ($\mathcal{D}_s^c$) until time s or, the error of fit until time s,

$$e_s = \left(\sum\nolimits_{x=0}^{\Omega} \mathbf{D}_s^c(x)\right) - \mathcal{D}_s^c. \tag{2}$$

We define time s as the number of days since the first observed death and T as the number of days since the first observed death from which the quarantine measures may start slowing the spread of the infection. Since the observed number of deaths until time T is subject to under- and over-reporting, we assume the following uninformative joint prior distribution on outputs

$$q_2(\Phi) = \begin{cases} 1 & \text{if } |e_T| < e_{max}, \\ 0 & \text{otherwise,} \end{cases} \tag{3}$$

where e_{max} is the maximum discrepancy of the model with respect to the observed number of deaths until time T. Therefore, we assume following [15] that the prior joint distribution of inputs and outputs are independent.

Since our goal is to obtain the joint posterior distribution of Θ, which we denote by $\pi^{[\Theta]}(\Theta)$, we need to update the joint prior distribution of inputs, $q_1(\Theta)$, using the observed data through a likelihood function on inputs and outputs, which we denote by $\mathcal{L}_1(\Theta)$ and $\mathcal{L}_2(\Phi)$, respectively. Because $M(\Theta)$ might not be invertible, we calculate the pooled joint prior distribution on outputs, denoted by $\tilde{q}^{[\Phi]}(\Phi)$, through geometric pooling[3]

$$\tilde{q}^{[\Phi]}(M(\Theta)) \propto q_1^*(M(\Theta))^{\alpha} q_2(M(\Theta))^{1-\alpha}, \tag{4}$$

where $q_1^*(M(\Theta))$ is the induced joint prior distribution of the outputs and α is the pooling weight. This is equivalent to finding the region on which both priors have common support [15]. A value of α close to one (resp. zero) will give a low (resp. high) weight of the information provided by the model on the posterior distribution of inputs. Thus, the Bayesian joint posterior distribution of the inputs is defined as

$$\pi^{[\Theta]}(\Theta) \propto \tilde{q}^{[\Phi]}(M(\Theta))\mathcal{L}_1(\Theta)\mathcal{L}_2(M(\Theta)). \tag{5}$$

To impose discipline in the inputs, we assume that the likelihood of retaining each temporal input $(x_1, x_2, x_3) = (\beta^{-1}, \delta^{-1}, \nu^{-1})$ follows a log normal distribution

$$\mathcal{L}_1(\theta) = \Pi_{i=1}^{3} \, \mathrm{P}(x_i|\theta), \text{ with } \log x_i \sim \mathcal{N}(\mu_{x_i}, \sigma_{x_i}^2). \tag{6}$$

The error e_s is distributed according to a Normal distribution with $\mu = 0$ and $\sigma = \sqrt{s}$, i.e., $\mathcal{N}(0, \sqrt{s})$.[4] Thus, we calculate the likelihood of retaining the set of parameters $\theta \in \Theta$ as

$$\mathcal{L}_2(M(\theta)) = \mathrm{P}(\{\mathcal{D}_s^c\}_{s=s_0}^{T}|\phi) = \Pi_{s=s_0}^{T} \, \mathrm{P}(\mathcal{D}_s^c|\phi) = \Pi_{s=s_0}^{T} (2\pi s)^{-1/2} \exp\left\{-e_s^2/(2s)\right\}, \tag{7}$$

where s_0 is the date at which the total number of deaths is above 50.

[3]Our model $M(\Theta)$ might not be invertible because several parameters are highly correlated.

[4]The error distribution of e_s is the result of assuming that the difference between the model's deaths and the observed deaths at any time t is i.i.d. according to a $\mathcal{N}(0, 1)$.

Model fitting. Similar to [13; 23] we fit the model to the evolution of the total number of deaths in the province of Hubei (China). For fitting purposes, we run our model assuming that the first COVID-19 case appeared on November 17, 2019 ($t = 1$), the first death occurred on January 11, 2020 ($t = 56$), and we stop the model when the deaths curve started to flatten on February 12, 2020 ($t = 88$), which is close to two weeks after the lockdown event on January 24, 2020 was introduced. Hence, we guarantee that our estimated parameters are not affected by non-pharmaceutical interventions. Note that in case of using other countries it is also necessary to take into account the date and number of imported cases and the date of introduction of non-pharmaceutical interventions. Thus, by focusing our analysis in the province of Hubei (China) we avoid having to calibrate more parameters and introducing more uncertainty in the model, which will lead to an unbearable computational burden. We use in the Bayesian melding data from day 71, when the total number of deaths overpassed 50 people, to day 88, when the total number of deaths were 1 114. The maximum discrepancy e_{max} is set at 150 deaths in order to allow for sufficient output variability. Moreover, we set the pooling weight, α, at 0.5 in order to give a similar importance to the model and the data.[5] Since the age distribution of the population in Hubei (China) resembles that of China, we scaled down the Chinese population to the total population size of Hubei. However, we keep the death tolls reported from official statistics, since the majority of the cases belong to Hubei.

To compute the likelihood of retaining each temporal input (i.e. $\mathcal{L}_1(\theta)$) given by Eq. (6), we assume, by using information from [5], that the expected times spent on each stage are distributed as

$$\log \beta^{-1} \sim \mathcal{N}(1.220, 0.85), \qquad \log \delta^{-1} \sim \mathcal{N}(1.570, 0.65), \qquad \log \nu^{-1} \sim \mathcal{N}(2.966, 0.50). \tag{8}$$

From (8) we have that the most likely period infecting individuals is 3.4 days, the average incubation period is 4.8 days, and the average time to recovery is 19.4 days. Other studies have also found larger incubation periods and infectious period for Covid-19. For instance, the [24]WHO's Covid-19 report 73 finds an average incubation period of 5-6 days and [22] finds an average duration of infectiousness of 8 days among patients with severe or critical Covid-19 symptoms.

Algorithm. The initial implementation of the model was done using the SIR algorithm of [18] with the priors as importance sample weights.[6] However, after generating 2 million samples with the SIR algorithm, the fraction of unique points was close to 6% with a resample of size 3 000. This is a signal that the posterior distribution $\pi^{[\Theta]}(\Theta)$ is concentrated near thin curve manifolds using the SIR algorithm. When this occurs, the number of samples that needs to be drawn from the original Bayesian melding will be overwhelmingly large. To avoid this problem, we follow the approach suggested by [17], in which they extend the incremental mixture importance sampling (IMIS) algorithm to the Bayesian melding. This algorithm has been applied before to epidemiological SIR models for analyzing the prevalence of HIV

[5] We also run the Bayesian melding assuming that $\alpha = 1$, which implies that we only give importance to the data. However, this case gives a worse fit to the data.

[6] See the preliminary results using the SIR algorithm in `https://www.medrxiv.org/content/10.1101/2020.04.29.20084400v1`

[7; 17]. Thus, the final sample is generated with the improved computational method called IMIS algorithm until the fraction of unique points reached 60% in the resample of size 3 000.[7]

Our analysis and calibration were performed with the use of Julia 1.3 (Julia Lab) and the visualization of the results with R software (R Foundation for Statistical Computing). Next, we detail the steps of the IMIS algorithm using geometric pooling: **IMIS algorithm**

(Raftery and Bao, 2010)

1. Initial Stage:

   (a) We use the initial 2 million samples (N_0) of θ values from the joint prior distribution on inputs $q_1(\Theta)$ obtained with the SIR algorithm

   (b) For each θ_i sampled, we run our SEIR model to obtain the output $M(\theta_i) = \phi_i$,

   (c) We estimate $q_1^*(\phi)$ using a standard gaussian kernel density estimator (kde)

   (d) We construct the importance sampling weights (ISW)

$$w_0(\theta_i) \propto \left(\frac{q_2(M(\theta_i))}{q_1^*(M(\theta_i))} \right)^{1-\alpha} \mathcal{L}_1(\theta_i)\mathcal{L}_2(M(\theta_i))$$

2. Importance Sampling Stage: for $k = 1, 2, \ldots,$ until a stopping criteria is satisfied

   (a) Compute a multivariate Gaussian distribution H_k with center $\theta^{(k)}$ and covariance $\Sigma^{(k)}$. Choose the maximum weight input as the center $\theta^{(k)}$. Calculate the weighted covariance matrix $\Sigma^{(k)}$ with the (B) 30 000 inputs with the smallest Mahalanobis distance to $\theta^{(k)}$ and the weights are the average between the importance weight and $1/N_k$.

   (b) Sample 30 000 new inputs θ_i from H_k and combine them with the previous ones

   (c) Compute steps 1(b)–(c) and calculate the new importance sampling weights as follows

$$w_k(\theta_i) \propto \left(\frac{q_2(M(\theta_i))}{q_1^*(M(\theta_i))} \right)^{1-\alpha} \mathcal{L}_1(\theta_i)\mathcal{L}_2(M(\theta_i)) \times \frac{q_1(\theta_i)}{q_1^{(k)}(\theta_i)},$$

   where $q_1^{(k)}(\theta_i)$ is the mixture sampling distribution, with $q_1^{(k)}(\theta_i) = \frac{N_0}{N_k} q_1(\theta_i) + \frac{B}{N_k} \sum_{s=1}^{k} H_s(\theta_i)$ and $N_k = N_0 + Bk$ is the total number of inputs up to iteration k.

3. Resample Stage: For J equal to 3 000, if the expected fraction of unique points after resampling $\hat{Q}(w) = \frac{1}{J} \sum_{i=1}^{N_k} (1 - (1 - w_i)^J)$ is less than 63%, go to Step 2.; otherwise, resample (J) 3 000 inputs with replacement from $\theta_1, \ldots, \theta_{N_K}$ with weights $w_1, \ldots, w_{N_K}$, where K is the number of iterations at step 2.

[7] We thank Adrian Raftery for suggesting the IMIS algorithm.

Results and validation. Figure S3 shows the fit of 3 000 SEIR models (blue lines), whose inputs were randomly drawn from $\pi^{[\Theta]}(\Theta)$, to the total number of deaths (black dots) in the province of Hubei (China). Despite the imperceptible difference during the first 88 days of the outbreak in the death toll across the 3 000 SEIR models, each SEIR model gives a quite different death toll after one year without any non-pharmaceutical intervention. In particular, we obtain that after one year ($t = 365$) the minimum death toll and the maximum death toll across the 3 000 SEIR models are 46 847 and 715 330 people, respectively, with a mean death toll of 352 152 and a standard deviation of 206 030. As a consequence, the estimated infection fatality rate will have wide credible intervals. This variance shows how complicated it is to pin down a unique model that is capable of explaining the current outbreak.

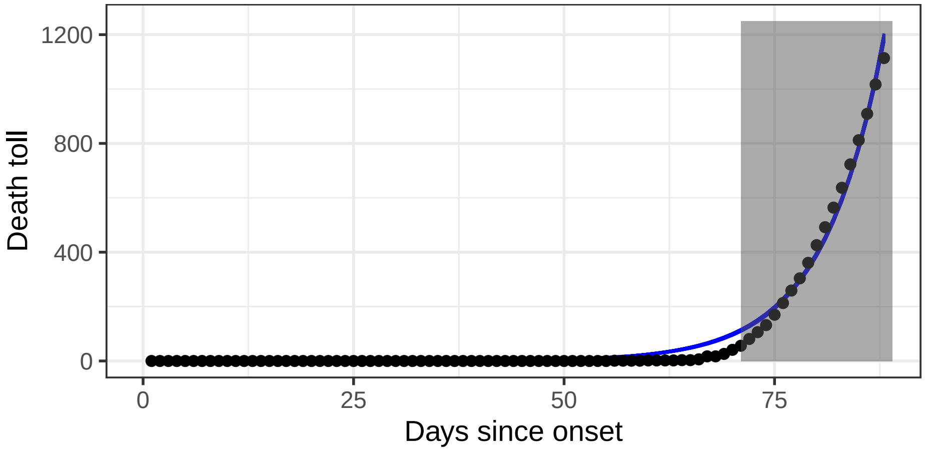

Figure S3: In-sample model performance of the SEIR models. Death toll in Hubei (China). *Notes:* Data (black dots). Simulation results (blue lines) based on 3000 random sets of parameter inputs drawn from the posterior distribution of $\pi^{[\Theta]}(\Theta)$. The gray area depicts the time interval used in the Bayesian melding.

The difficulty of fitting a single model to the existing data can also be seen looking at the correlation matrix shown in Figure S4. In particular, Fig. S4 shows that the parameters are highly correlated. Each dot corresponds to a random draw of θ (or one SEIR model) from $\pi^{[\Theta]}(\Theta)$. Thus, Fig. S4 shows that the effective infectious rate, β, and the removal rate, ν, are close to be perfectly correlated (.935). This means that higher removal rates or, equivalently, shorter recovery periods are associated with higher COVID-19 transmission rates. Similarly, the removal rate, ν, and the fatality adjustment factor, ε, are negative strongly correlated (-.856). This means that higher adjustment factors, which imply a higher infection fatality rate, are associated with longer recovery periods.

Figure S5 summarizes the marginal posterior distributions of the model inputs (Θ). The top panel shows through a series of boxplots the marginal posterior distribution of the infection fatality rate at each age. The bottom panels show the marginal posterior distribution of the effective transmission rate, β, the removal rate, ν, and the inverse of the incubation period, δ. Table S1 summarizes the period that an individual spends in each stage. Notice that since underreported infected cases are comprised of symptomatic and asymptomatic

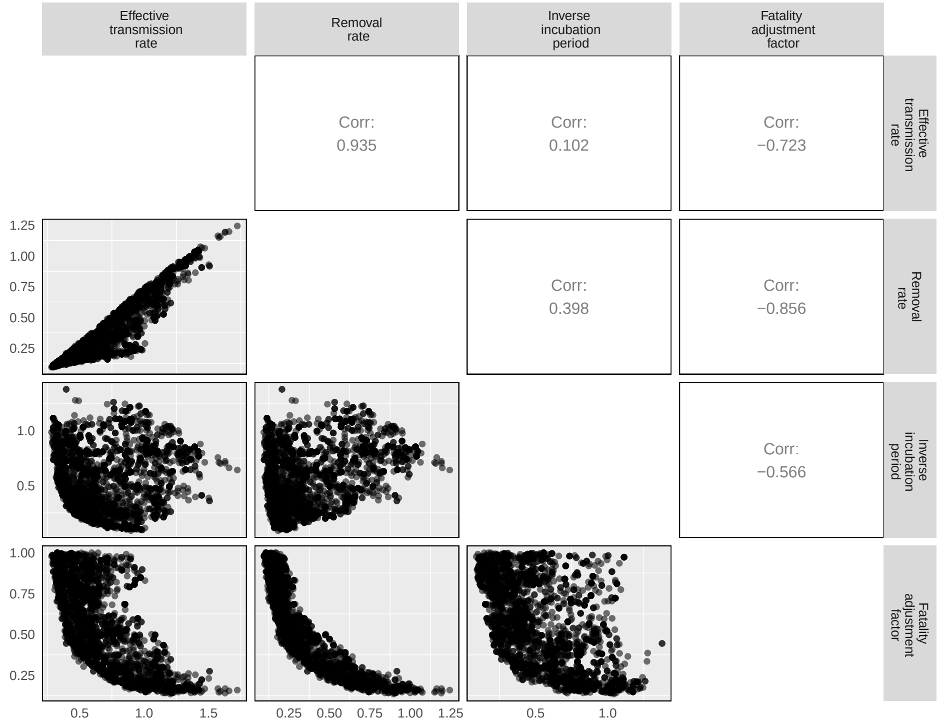

Figure S4: Correlation matrix of the SEIR model inputs based on the posterior distribution of $\pi^{[\Theta]}(\Theta)$.

individuals, the average periods are shorter than those usually reported for symptomatic individuals [see 5], which we used as likelihoods. This effect can be observed in Fig. S4 through the negative correlation between the fatality adjustment factor and all the three other parameters, especially for the removal rate. Parameters β^{-1}, δ^{-1}, and ν^{-1} measure the average period that an individual spends infecting other people, in incubation, and recovering, respectively. We obtain that the estimated incubation period is 2.57 (0.92-7.85, 95% CI) days. The terms in parenthesis show the 95 percent credible intervals. The estimated recovery period is on average 4.12 (1.05-8.32, 95% CI) days and the estimated transmission period is on average 1.7 (0.75-3.07, 95% CI) days. Adding the incubation period and the recovery period, we estimate that the period from being infected to recovered is 6.7 (1.97-16.17, 95% CI) days. Notice that these three average durations are shorter than those used in (8) from [5] as well as those reported by [22]. This is because the model accounts for both symptomatic and asymptomatic individuals, regardless whether they have been detected or not. The estimated infection fatality rate is close to half of the underlying case fatality rate $\mathbf{m}^c$ estimated using data from [19], and with a probability of 95 percent we estimate that the infection fatality rate is between 16% and 98% the case fatality rate $\mathbf{m}^c$.

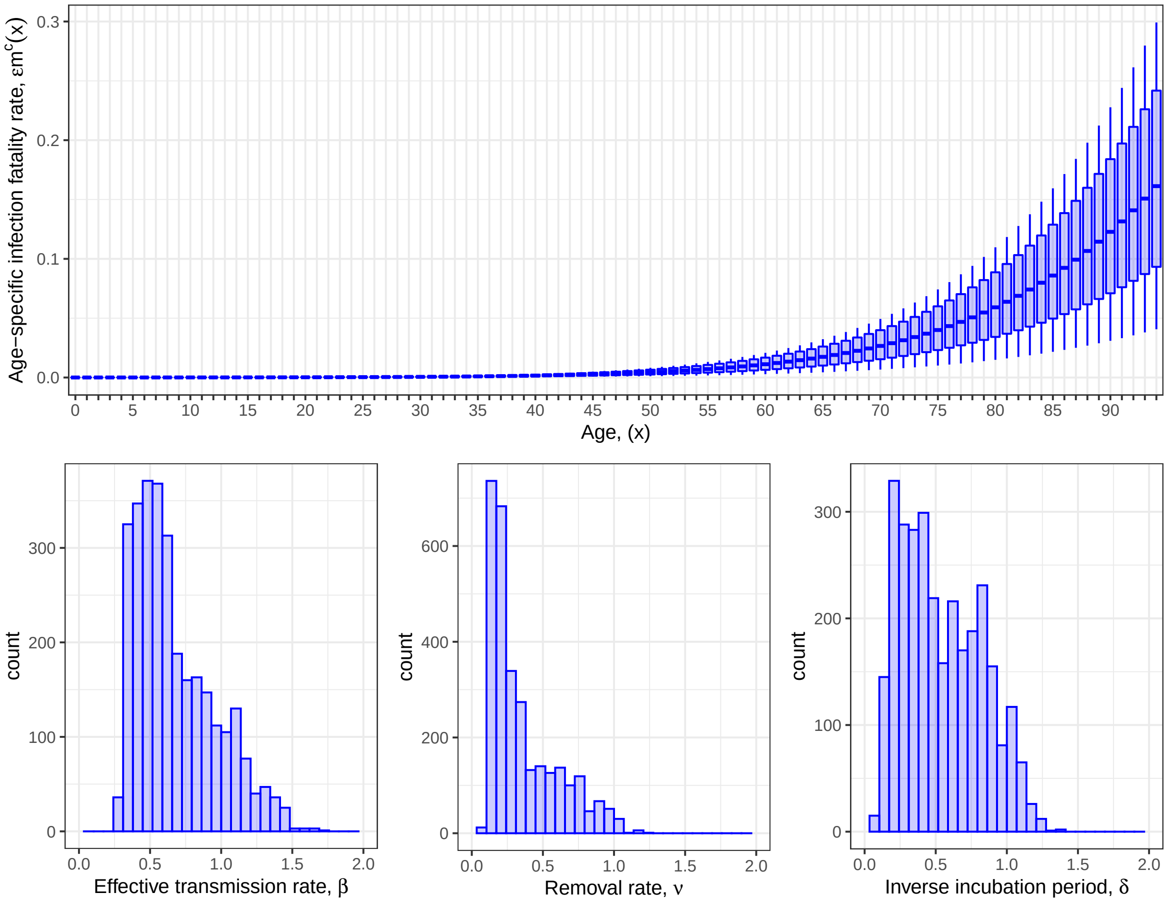

Figure S5: Posterior distribution of the SEIR model inputs $\pi^{[\Theta]}(\Theta)$.

Table S1: The epidemic parameters of the SEIR model

	Parameter	Mean	2.5 pct	Median	97.5 pct
Transmission (days)	β^{-1}	1.74	0.75	1.69	3.07
Incubation (days)	δ^{-1}	2.57	0.92	2.07	7.85
Recovery (days)	ν^{-1}	4.12	1.05	3.73	8.32
Fatality adjustment	ε	0.56	0.16	0.54	0.98

S2 Infection fatality rates

From the sampled inputs generated from the posterior distribution $\pi^{[\Theta]}(\Theta)$, we run the same 3 000 SEIR models for each U.S. state for a period of a year ($t = 365$). The output that we will report in this section is the infection fatality rate (the ratio between the total deaths to total infected people) after a year ($t = 365$), so that we guarantee that in all simulations

Table S2: Estimated infection fatality rates (IFR) in U.S. (in %)

#	Country	IFR	90% CI	68% CI	#	Country	IFR	90% CI	68% CI
1	Alabama	1.02	(0.32, 1.76)	(0.43, 1.59)	27	Montana	1.10	(0.34, 1.91)	(0.47, 1.73)
2	Alaska	0.75	(0.23, 1.30)	(0.32, 1.18)	28	Nebraska	0.99	(0.31, 1.71)	(0.42, 1.55)
3	Arizona	1.05	(0.33, 1.81)	(0.44, 1.64)	29	Nevada	0.93	(0.29, 1.60)	(0.39, 1.45)
4	Arkansas	1.02	(0.32, 1.77)	(0.43, 1.60)	30	New Hampshire	1.11	(0.35, 1.92)	(0.47, 1.74)
5	California	0.92	(0.29, 1.59)	(0.39, 1.44)	31	New Jersey	1.04	(0.32, 1.80)	(0.44, 1.63)
6	Colorado	0.87	(0.27, 1.51)	(0.37, 1.37)	32	New Mexico	1.05	(0.33, 1.81)	(0.44, 1.64)
7	Connecticut	1.11	(0.34, 1.92)	(0.47, 1.74)	33	New York	1.06	(0.33, 1.83)	(0.45, 1.65)
8	Delaware	1.12	(0.35, 1.93)	(0.47, 1.75)	34	North Carolina	0.99	(0.31, 1.71)	(0.42, 1.55)
9	District of Columbia	0.80	(0.25, 1.39)	(0.34, 1.25)	35	North Dakota	1.00	(0.31, 1.73)	(0.43, 1.57)
10	Florida	1.25	(0.39, 2.16)	(0.53, 1.96)	36	Ohio	1.05	(0.33, 1.81)	(0.44, 1.64)
11	Georgia	0.86	(0.27, 1.48)	(0.36, 1.34)	37	Oklahoma	0.96	(0.30, 1.66)	(0.41, 1.50)
12	Hawaii	1.21	(0.37, 2.08)	(0.51, 1.89)	38	Oregon	1.04	(0.32, 1.80)	(0.44, 1.63)
13	Idaho	0.94	(0.29, 1.62)	(0.40, 1.46)	39	Pennsylvania	1.14	(0.35, 1.96)	(0.48, 1.78)
14	Illinois	0.99	(0.31, 1.72)	(0.42, 1.55)	40	Rhode Island	1.10	(0.34, 1.91)	(0.47, 1.72)
15	Indiana	0.97	(0.30, 1.67)	(0.41, 1.51)	41	South Carolina	1.04	(0.32, 1.80)	(0.44, 1.63)
16	Iowa	1.07	(0.33, 1.85)	(0.45, 1.68)	42	South Dakota	1.04	(0.32, 1.80)	(0.44, 1.63)
17	Kansas	1.01	(0.31, 1.74)	(0.43, 1.57)	43	Tennessee	0.98	(0.30, 1.70)	(0.42, 1.54)
18	Kentucky	0.99	(0.31, 1.71)	(0.42, 1.55)	44	Texas	0.79	(0.25, 1.37)	(0.34, 1.24)
19	Louisiana	0.95	(0.29, 1.64)	(0.40, 1.48)	45	Utah	0.69	(0.21, 1.19)	(0.29, 1.08)
20	Maine	1.23	(0.38, 2.13)	(0.52, 1.93)	46	Vermont	1.16	(0.36, 2.01)	(0.49, 1.82)
21	Maryland	0.98	(0.30, 1.70)	(0.42, 1.53)	47	Virginia	0.97	(0.30, 1.68)	(0.41, 1.52)
22	Massachusetts	1.05	(0.32, 1.81)	(0.44, 1.63)	48	Washington	0.94	(0.29, 1.63)	(0.40, 1.47)
23	Michigan	1.05	(0.33, 1.82)	(0.45, 1.64)	49	West Virginia	1.17	(0.36, 2.02)	(0.50, 1.83)
24	Minnesota	1.00	(0.31, 1.73)	(0.43, 1.57)	50	Wisconsin	1.05	(0.33, 1.82)	(0.45, 1.64)
25	Mississippi	0.97	(0.30, 1.67)	(0.41, 1.51)	51	Wyoming	1.00	(0.31, 1.74)	(0.43, 1.57)
26	Missouri	1.04	(0.32, 1.81)	(0.44, 1.63)					

Notes: 90% and 68% credible intervals are reported inside the parenthesis.

all people infected are either recovered or dead. Notice that given that the age distribution of the population differ in each state and we account for the differential mortality by age of COVID-19, the total number of deaths from COVID-19 will not coincide across states, if the number of people infected coincides across states.

Table S2 shows the estimated infection fatality rate (IFR) for 51 states in the U.S.. Population counts by age and age-specific mortality rates are taken for each state from [21].

The state with the highest estimated IFR is Florida with 1.25% (0.39-2.16%, 90% CI) followed by Maine 1.23% (0.38-2.13%, 90% CI), Hawaii 1.21% (0.37-2.08%, 90% CI), West Virginia 1.17% (0.36-2.02%, 90% CI), and Vermont 1.16% (0.36-2.01%, 90% CI). The state with the lowest estimated IFR is Utah with 0.69% (0.21-1.19%, 90% CI), followed in reverse order by Alaska 0.75% (0.23-1.30%, 90% CI), Texas 0.79% (0.25-1.37%, 90% CI), District of Columbia 0.80% (0.25-1.39%, 90% CI), and Georgia 0.86% (0.27-1.48%, 90% CI). The terms in parenthesis show the 90 and 68 percent credible intervals, which are the intervals within which the IFR for each state falls with a probability of 90 and 68 percent, respectively. The most important demographic factor explaining the variation in IFRs across countries is the mean-age of the population, as it is shown by [20]. Thus, with a probability of 90 percent, we obtain that the estimated IFR ranges between 0.36 and 2.16 percent in the most aged states

and between 0.21 and 1.48 percent in the less aged states. With a probability of 68 percent, the estimated IFR ranges between 0.49 and 1.96 percent in the most aged states and between 0.29 and 1.34 percent in the less aged states. In the next subsections we explain how we can combine the estimated IFR reported in Table S2 and the epidemiological information reported by each state to indirectly estimate the fraction of people ever infected and the fraction of people detected among the ever infected.

S3 Fraction of people ever infected

In this section we indirectly estimate the fraction of people ever infected from COVID-19 across U.S. states. To do so, we make use of the definition of the infection fatality rate or the ratio between the total number of deaths from COVID-19 and the total number of people infected until time t. Equation (9) shows that the total number of infected people (detected and undetected) is given by the probability of ever being infected among the total susceptible population until time t

$$\text{IFR} = \frac{\text{Total deaths}_t}{\text{Total infected}_t} = \frac{\text{Total deaths}_t}{\text{Population}_t \times \text{Pr}\begin{pmatrix}\text{being} \\ \text{infected}_t\end{pmatrix}}. \tag{9}$$

From (9) we can indirectly calculate the fraction of people ever infected or the probability of ever being infected until time t as

$$\begin{matrix}\text{Fraction of people} \\ \text{ever infected}_t\end{matrix} = \frac{\text{Total deaths}_t}{\text{Population}_t \times \text{IFR}}. \tag{10}$$

An important feature of our indirectly estimated fraction of people ever infected is that it depends on the total number of observed deaths, see the right-hand side of (10). Therefore, it is not necessary that our model accounts for the evolution of the total number of infected people at each time t, which validates our simulation strategy.

External validation. Previous to estimating the fraction of people ever infected across U.S. states, we show that our indirect estimation method is capable of replicating existing studies of seroprevalence and fraction of people ever infected across different regions and countries. We focus our analysis on seroprevalence studies that include all age groups of the population. In particular, we use the first rounds of seroprevalence studies in Brazil, Spain and those conducted for New York City (NYC) and the states of Connecticut, Missouri, and New York [10; 11; 14]. Figure S6 shows that our indirect estimation replicates well the fraction of people ever infected among the total population reported in these seroprevalence studies. Panel **A** compares the observed fraction of people ever infected (horizontal axis) to our estimated fraction of people ever infected (vertical axis). The diagonal line is the 45 degree line. The dot at the top-right corner depicts the fraction of people ever infected in NYC. The two more distant green dots from the 45 degree line correspond to two adjacent regions in Spain (La Rioja and Navarra). Red dots present a higher dispersion relative to the seroprevalence studies in Spain and U.S. due to the smaller sample size of each seroprevalence study in Brazil [see 10; 11; 14]. In Brazil, despite individuals and households being randomly selected, the amount of surveys conducted were the same across all cities (between 200 and

250), irrespective of population size. Hence, extrapolating from a sample of 250 individuals to the city level in a place like São Paulo with 12.3 million inhabitants could be affecting the results by increasing the uncertainty levels. Panel **B** shows that the distribution of the errors (i.e. the difference between the observed and the estimated fraction of people ever infected) is almost centered at zero, with a small bias towards underreporting the observed fraction of people ever infected. Panels **C** and **D** show the absolute and relative errors as a function of the observed fraction ever infected. The mean absolute error and the mean relative error are -0.16% and 0.095, respectively. Therefore, Figure S6 validates our approach. See actual numbers in Table S5 in Section S5. In addition, figures S7 and S8 in Section S5 compare the model results to the first and fourth rounds of the "Nationwide Commercial Laboratory Seroprevalence Survey" reported by the CDC.

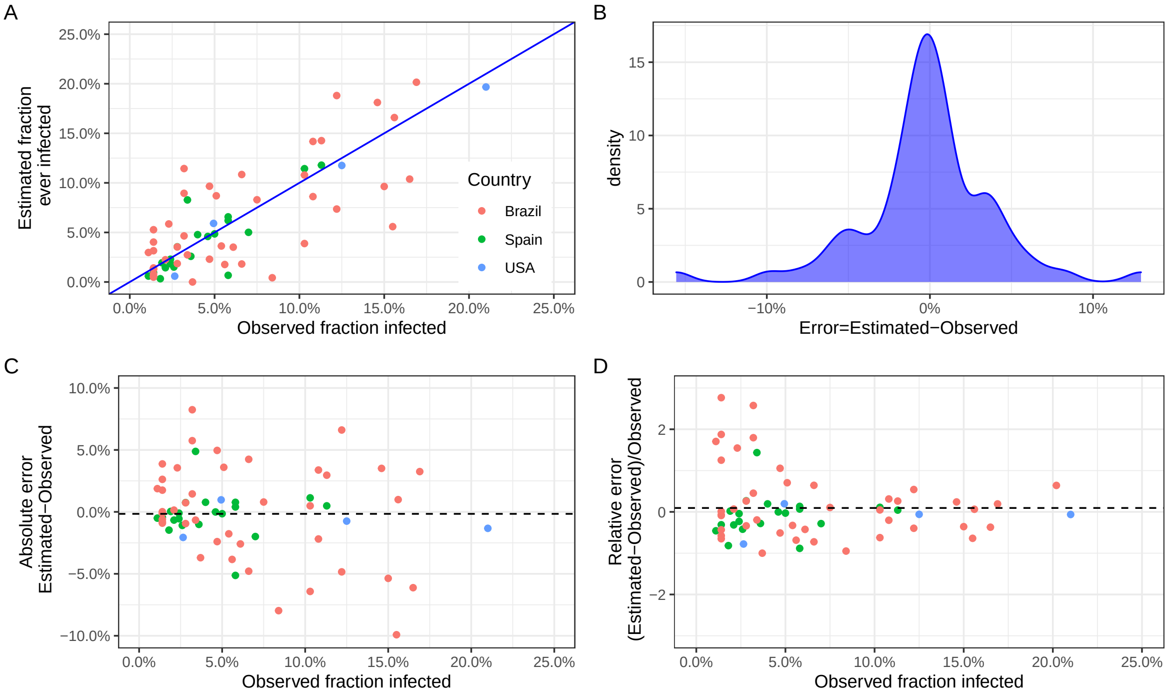

Figure S6: Observed vs. estimated fraction of people ever infected. Panels **A, B,** and **C** show the absolute error in three different formats, while Panel **D** shows the relative error. *Notes*: Dashed black lines show the mean error value. The observed data includes information from seroprevalence studies conducted in 67 regions: 19 regions in Spain (green dots), New York City, the states of Connecticut, Missouri, and New York (blue dots), and 44 regions in Brazil (red dots).

Results. After showing that our approach is capable of replicating existing seroprevalence studies, we apply our model to estimate the fraction of people ever infected across U.S. states. The states analyzed satisfy two criteria. First, the state has a stable death toll. We assume that a stable death toll is reached when its average rate of growth during the last month is less than 0.5%. This condition guarantees that most infected people are either recovered or dead. Second, we exclude states with less than 500 reported deaths from COVID-19 in

Table S3: Estimated fraction of people ever infected in selected U.S. states (in %): Ranked according to the fraction of people ever infected

#	State	Population	Deaths	Fraction infected	90% CI	68% CI	Date
1	New Jersey	8 872 797	15 996	17.3	(10.0, 55.8)	(11.1, 40.8)	09.08.20
2	New York	19 428 618	33 016	16.1	(9.3, 51.9)	(10.3, 38.0)	09.08.20
3	Massachusetts	6 887 571	9 141	12.7	(7.3, 40.9)	(8.1, 29.9)	09.08.20
4	Connecticut	3 558 382	4 474	11.3	(6.6, 36.5)	(7.2, 26.7)	09.08.20
5	District of Columbia	701 570	611	10.9	(6.3, 35.0)	(6.9, 25.6)	09.08.20
6	Rhode Island	1 054 930	1 059	9.1	(5.3, 29.3)	(5.8, 21.5)	09.08.20
7	Louisiana†	4 632 867	3 610	8.2	(4.8, 26.5)	(5.3, 19.4)	07.21.20
8	Illinois	12 644 110	8 405	6.7	(3.9, 21.6)	(4.3, 15.8)	09.08.20
9	Michigan	9 983 147	6 811	6.5	(3.8, 20.9)	(4.2, 15.3)	09.08.20
10	Maryland	6 010 630	3 807	6.5	(3.7, 20.8)	(4.1, 15.2)	09.08.20
11	Delaware	970 344	609	5.6	(3.2, 18.1)	(3.6, 13.2)	09.08.20
12	Pennsylvania	12 795 687	7 780	5.4	(3.1, 17.2)	(3.4, 12.6)	09.08.20
13	Indiana	6 729 379	3 380	5.2	(3.0, 16.7)	(3.3, 12.2)	09.08.20
14	Colorado	5 722 795	1 973	3.9	(2.3, 12.7)	(2.5, 9.3)	09.08.20
15	Ohio†	11 680 342	4 259	3.5	(2.0, 11.2)	(2.2, 8.2)	09.06.20
16	Minnesota	5 637 493	1 914	3.4	(2.0, 10.9)	(2.2, 8.0)	09.08.20
17	Virginia	8 420 245	2 686	3.3	(1.9, 10.6)	(2.1, 7.8)	09.08.20
18	Washington	7 562 699	1 953	2.7	(1.6, 8.8)	(1.8, 6.5)	09.08.20
19	Iowa†	3 153 268	789	2.3	(1.4, 7.5)	(1.5, 5.5)	07.18.20
20	Wisconsin †	5 819 432	893	1.5	(0.8, 4.7)	(0.9, 3.4)	07.27.20

Source: Authors' calculations using data from COVID-19 Data Repository by the Center for Systems Science and Engineering (CSSE) at Johns Hopkins University on September 8, 2020. *Notes:* 90% and 68% credible intervals are reported in the sixth and seventh columns. The last column reports for each state the latest date at which all the requirements for this calculation are satisfied. † Notice that the estimated result could be altered if the data available by September 8, 2020 is used.

order to guarantee that COVID-19 deaths are distributed across most age groups. This last criteria excludes 15 states (Alaska, Hawaii, Idaho, Kansas, Maine, Montana, Nebraska, New Hampshire, North Dakota, Oregon, South Dakota, Utah, Vermont, West Virginia, Wyoming) in which less than 500 COVID-19 deaths have been reported. As a result, by combining epidemiological data taken from the COVID-19 Data Repository by the Center for Systems Science and Engineering (CSSE) at Johns Hopkins University on September 8, 2020 and the distribution of IFR estimated with our model, we report in Table S3 the fraction of people ever infected among the total population for 20 states.

Table S3 shows that the state with the highest estimated fraction of people ever infected is New Jersey with 17.3% (10.0-55.8, 90% CI), followed by New York 16.1% (9.3-51.9, 90% CI), Massachusetts 12.7% (7.3-40.9, 90% CI), Connecticut 11.3% (6.6-36.5, 90% CI), and the District of Columbia 10.9% (6.3-35.0, 90% CI). The last column of Table S3 shows the date at which this fraction is estimated. Nonetheless, since the indirect estimation of the fraction of people ever infected depends on the accuracy of the total number of deaths from COVID-19,

Table S3 should be read cautiously. If there is a state or country that has systematically excluded (included) a fraction d of the true total deaths from COVID-19 (or included non COVID-19 deaths), the true fraction of people infected will be d times higher (lower) than the fraction of people ever infected reported in Table S3. Thus, for instance, if the total excess deaths in Spain during the period March 4th to August 25th are hypothetically COVID-19 related deaths, the estimated fraction of people ever infected in Spain using our approach should be increased almost by fifty five percent $d = 1,55 = \frac{44\,448}{28\,754}$, where 44 448 is the total excess death and 28 754 are the total COVID-19 deaths reported by August 25th. Thereby, the estimated fraction of people ever infected in Spain would hypothetically increase from 4.9 percent to 7.6 percent.[8] Similarly, if a better treatment is found, which reduces by X percent the IFR, our estimated fraction of people ever infected would increase by X percent.

S4 Fraction of people detected among the ever infected

In this section we estimate the fraction of ever infected people who are detected or the probability of being detected among the ever infected. This calculation is useful for assessing the effectiveness of testing policies and also for detecting regions or groups that need additional testing. To do so, we use the definition of the infection fatality rate (IFR) and the case fatality rate (CFR). The CFR is the ratio between the total number of deaths from COVID-19 and the total number of infected cases detected. The total number of people infected who are detected differs from the total number of infected cases, used in the definition of IFR, in that these people have been detected through a test. Thus, the CFR is given by

$$\text{CFR} = \frac{\text{Total deaths}_t}{\begin{array}{c}\text{Total infected}\\ \text{and detected}_t\end{array}} = \frac{\text{Total deaths}_t}{\text{Population}_t \times \Pr\left(\begin{array}{c}\text{being}\\ \text{infected}_t\end{array}\right) \times \Pr\left(\begin{array}{c}\text{being}\\ \text{detected}_t\end{array}\middle|\begin{array}{c}\text{being}\\ \text{infected}_t\end{array}\right)}. \tag{11}$$

We do not include a time subscript in CFR in order to stress that the epidemiological data collected should be stable. Dividing (9) by (11), we get that the fraction of people detected among the ever infected or the probability of being detected among the ever infected until time t is

$$\begin{array}{c}\text{Fraction}\\ \text{detected}_t\end{array} = \frac{\text{Total infected and detected}_t}{\text{Total infected}_t} = \frac{\text{IFR}}{\text{CFR}}. \tag{12}$$

Therefore, by combining the estimated IFR and the CFR for each region, we can indirectly estimate the fraction of people detected among the ever infected. It should be kept in mind that this measure gives information about the strength and effectiveness detecting infected cases since the beginning of the outbreak but, it does not represent well the current fraction of people detected among the infected.

We show in Table S4 the estimated fraction of people who are detected among the ever infected from COVID-19 for the same 20 U.S. states reported in Table S3. Table S4 shows that the state with the highest estimated fraction of individuals detected among the ever infected is Wisconsin 58.2% (18.1-100.0, 90% CI), followed by Iowa with 52.0% (16.9-90.0,

[8] See, for instance, the "tracking covid-19 excess deaths across countries" at the Economist, https://www.economist.com/graphic-detail/2020/07/15/tracking-covid-19-excess-deaths-across-countries.

90% CI), Virginia 46.3% (14.4-80.1, 90% CI), Minnesota 42.7 (13.3-73.9, 90% CI), and Washington 37.4% (11.6-64.7, 90% CI). The last column of Tab. S4 reports the date used for the estimated fraction of people detected among the ever infected across the 20 states. According to Table S4, except for the first eight states, we observe with a probability of 90 percent that the fraction of detected people among the ever infected has been less than 50% since the beginning of the epidemic. For these states this result implies that, unless that other non-pharmaceutical interventions such as social distancing, the use of masks, and hygiene measures are implemented, it is expected that the spread of the virus will continue.

Table S4: Estimated fraction of ever infected individuals who are detected in the U.S. (in %): Ranked according to the fraction of people detected.

#	State	IFR	CFR	Fraction detected	90% CI	68% CI	Date
1	Wisconsin†	1.05	1.81	58.2	(18.1, 100.0)	(24.7, 91.0)	07.27.20
2	Iowa†	1.07	2.06	52.0	(16.2, 90.0)	(22.1, 81.4)	07.18.20
3	Virginia	0.97	2.09	46.3	(14.4, 80.1)	(19.6, 72.4)	09.08.20
4	Minnesota	1.00	2.35	42.7	(13.3, 73.9)	(18.1, 66.9)	09.08.20
5	Washington	0.94	2.52	37.4	(11.6, 64.7)	(15.9, 58.5)	09.08.20
6	Delaware	1.12	3.33	33.6	(10.4, 58.1)	(14.2, 52.6)	09.08.20
7	Ohio†	1.05	3.26	32.2	(10.0, 55.6)	(13.6, 50.3)	09.06.20
8	Illinois	0.99	3.31	30.1	(9.3, 52.0)	(12.7, 47.0)	09.08.20
9	Maryland	0.98	3.36	29.2	(9.1, 50.5)	(12.4, 45.7)	09.08.20
10	Indiana	0.97	3.35	28.9	(9.0, 49.9)	(12.2, 45.2)	09.08.20
11	Colorado	0.87	3.31	26.4	(8.2, 45.7)	(11.2, 41.4)	09.08.20
12	Louisiana†	0.95	3.74	25.4	(7.9, 43.9)	(10.8, 39.7)	07.21.20
13	Rhode Island	1.10	4.69	23.5	(7.3, 40.7)	(10.0, 36.8)	09.08.20
14	Pennsylvania	1.14	5.36	21.2	(6.6, 36.6)	(9.0, 33.1)	09.08.20
15	District of Columbia	0.80	4.25	18.8	(5.8, 32.6)	(8.0, 29.5)	09.08.20
16	Michigan	1.05	5.73	18.3	(5.7, 31.7)	(7.8, 28.7)	09.08.20
17	New York	1.06	7.49	14.1	(4.4, 24.4)	(6.0, 22.0)	09.08.20
18	Massachusetts	1.05	7.43	14.1	(4.4, 24.3)	(6.0, 22.0)	09.08.20
19	Connecticut	1.11	8.32	13.3	(4.1, 23.1)	(5.7, 20.9)	09.08.20
20	New Jersey	1.04	8.22	12.7	(3.9, 21.9)	(5.4, 19.8)	09.08.20

Source: Authors' calculations using data from COVID-19 Data Repository by the Center for Systems Science and Engineering (CSSE) at Johns Hopkins University on September 8, 2020. *Notes:* 90% and 68% credible intervals are reported in the sixth and seventh columns. The last column reports for each state the latest date at which all the requirements for this calculation are satisfied. † Notice that the estimated result could be altered if the data available by September 8, 2020 is used.

References

[1] L. Bao, Raftery, A.E., A stochastic infection rate model for estimating and projecting national HIV prevalence rates. *Sexually Transmitted Infections* **86** (2010): i93–i99.

[2] L. Bao, Raftery, A.E., Reddy, A., Estimating the sizes of populations at risk of HIV infection in Bangladesh using a Bayesian hierarchical model. *Statistics and Its Interface*, **8** (2015): 125–136.

[3] L. Bao, Salomon, J.A., Brown, T., Raftery, A.E., Hogan, D., Modeling HIV/AIDS epidemics: revised approach in the UNAIDS Estimation and Projection Package 2011. *Sexually Transmitted Infections* **88** (2012): i3–i10.

[4] L. Bao et al., Modelling national HIV/AIDS epidemics: revised approach in the UNAIDS Estimation and Projection Package 2011. *Sexually Transmitted Infections* **88**. Suppl 2 (2012): i3–i10.

[5] Bi, Qifang, et al. Epidemiology and transmission of COVID-19 in 391 cases and 1286 of their close contacts in Shenzhen, China: A retrospective cohort study. *The Lancet Infectious Diseases* (2020).

[6] T. Brown, Bao, L., Raftery, A.E., Salomon, J.A., Baggaley, R.F., Stover, J., Gerland P., Modelling HIV epidemics in the antiretroviral era: The UNAIDS Estimation and Projection package 2009. *Sexually Transmitted Infections* **86** (2010): i3–i10.

[7] S. J. Clark, Thomas, J. R., Bao, L.,Estimates of age-specific reductions in HIV prevalence in Uganda: Bayesian melding estimation and probabilistic population forecast with an HIV-enabled cohort component projection model. *Demographic Research* **27** (2012).

[8] J. B. Dowd, V. Rotondi, L. Adriano, D. M. Brazel, P. Block, X. Ding, ... , M. C. Mills, Demographic science aids in understanding the spread and fatality rates of COVID-19. *Proceedings of the National Academy of Sciences*, **117** 18 (2020): 9696–9698.

[9] J. R. Goldstein, Lee, R. D., Demographic Perspectives on Mortality of Covid-19 and Other Epidemics. *Proceedings of the National Academy of Sciences of the United States of America*, (2020). doi: `https://doi.org/10.1073/pnas.2006392117`

[10] P. Hallal, et al., Remarkable variability in SARS-CoV-2 antibodies across Brazilian regions: nationwide serological household survey in 27 states. medRxiv, 2020.05.30.20117531 (2020).

[11] F. P. Havers, et al., Seroprevalence of antibodies to SARS-CoV-2 in six sites in the United States, March 23-May 3, 2020. *JAMA Internal Medicine*, Published online July 21, 2020. doi: `10.1001/jamainternmed.2020.4130`

[12] A. Lachmann, K. M. Jagodnik, F. M. Giorgi, F. Ray, Correcting under-reported COVID-19 case numbers: estimating the true scale of the pandemic. *medRxiv*, (2020). doi: `https://doi.org/10.1101/2020.03.14.20036178`

[13] G. Marois, R. Muttarak, S. Scherbov, Assessing the potential impact of COVID-19 on life expectancy. *PloS one* 15.9 (2020): e0238678. https://dx.plos.org/10.1371/journal.pone.0238678

[14] M. Pollán, et al., Prevalence of SARS-CoV-2 in Spain (ENE-COVID): a nationwide, population-based seroepidemiological study. *Lancet* (2020) https:/doi.org/10.1016/S0140-6736(20)31483-5.

[15] D. Poole, A. E. Raftery, Inference for deterministic simulation models: the Bayesian melding approach. *Journal of the American Statistical Association*, **95**(452), 1244–1255, (2000).

[16] Report of the WHO-China Joint Mission on Coronavirus Disease 2019 (COVID-19) [Pdf] - World Health Organization, Feb. 28, (2020).

[17] A. E. Raftery, Bao, L., Estimating and projecting trends in HIV/AIDS generalized epidemics using incremental mixture importance sampling. *Biometrics*, **66** (2010): 1162–1173.

[18] D. B. Rubin, The calculation of posterior distributions by data augmentation: Comment: A non iterative sampling/importance resampling alternative to the data augmentation algorithm for creating a few imputations when fractions of missing information are modest: The SIR algorithm. *Journal of the American Statistical Association* **82** (1987): 543–546.

[19] V. Surveillances, The epidemiological characteristics of an outbreak of 2019 novel Coronavirus diseases (COVID-19)? China, 2020. *China CDC Weekly*, **2**(8), 113-122, (2020).

[20] M. Sánchez-Romero, di Lego, V., Prskawetz, A., Queiroz, B. L., How many lives can be saved? A global view on the impact of testing, fraction of people infected and demographics on COVID-19 fatality rates. medRxiv 2020.04.29.20084400; doi: https://doi.org/10.1101/2020.04.29.20084400

[21] United States Mortality DataBase. University of California, Berkeley (USA). Available at www.usa.mortality.org (data downloaded on 06.10.2020)

[22] van Kampen, J. J.A., et al, 2020. Shedding of infectious virus in hospitalized patients with coronavirus disease-2019 (COVID-19): duration and key determinants. medRxiv 2020.06.08.20125310; doi: https://doi.org/10.1101/2020.06.08.20125310

[23] R. Verity, L. C. Okell, I. Dorigatti, P. Winskill, C. Whittaker, N. Imai, ..., A. Dighe, Estimates of the severity of coronavirus disease 2019: a model-based analysis. *The Lancet Infectious Diseases*, (2020).

[24] World Health Organization. Coronavirus disease 2019 (COVID-19). Situation Report 73, (2020). https://www.who.int/docs/default-source/coronaviruse/situation-reports/20200402-sitrep-73-covid-19.pdf?sfvrsn=5ae25bc7_2

S5 Additional tables and figures

In this section we show in Table S5 the numbers plotted in Figure S6, see Section S3, and complement our external validation by comparing in figures S7 and S8 our estimated fraction of people ever infected with the first and last nationwide seroprevalence studies carried out in the U.S..

Table S5: Observed vs. estimated fraction of people ever infected (in %)

Country	State/Province/City	Population	Positives	Deaths	Fraction infected	90% CI	68% CI	Seroprevalence study	Error	
									Absolute	Relative
Spain	España	46 977 305	277 719	27 650	4.85	(2.81, 15.63)	(3.1, 11.44)	5	-0.15	-0.03
Spain	Andalucía	8 432 967	16 432	1 358	1.51	(0.87, 4.86)	(0.96, 3.56)	2.6	-1.09	-0.42
Spain	Aragón	1 319 290	7 177	838	4.59	(2.66, 14.79)	(2.94, 10.83)	4.6	-0.01	0
Spain	Asturias	1 016 129	3 344	315	1.94	(1.12, 6.24)	(1.24, 4.57)	1.9	0.04	0.02
Spain	Cantabria	579 925	2 773	207	2.59	(1.5, 8.34)	(1.66, 6.11)	3.6	-1.01	-0.28
Spain	Ceuta	84 335	175	4	0.6	(0.34, 1.92)	(0.38, 1.41)	1.1	-0.5	-0.46
Spain	Castilla y León	2 390 570	24 696	1 946	5.01	(2.9, 16.13)	(3.2, 11.81)	7	-1.99	-0.28
Spain	Castilla-La Mancha	2 032 321	24 908	2 893	11.43	(6.61, 36.82)	(7.31, 26.96)	10.3	1.13	0.11
Spain	Canarias	2 216 667	2 289	151	0.67	(0.39, 2.17)	(0.43, 1.59)	5.8	-5.13	-0.88
Spain	Catalunya	7 589 198	57 467	5 944	6.57	(3.8, 21.17)	(4.2, 15.5)	5.8	0.77	0.13
Spain	Extremadura	1 060 071	3 948	500	3.55	(2.05, 11.44)	(2.27, 8.37)	2.8	0.75	0.27
Spain	Galicia	2 687 926	10 960	606	1.44	(0.83, 4.63)	(0.92, 3.39)	2.1	-0.66	-0.32
Spain	Islas Baleares	1 196 441	2 096	218	1.85	(1.07, 5.95)	(1.18, 4.36)	2.4	-0.55	-0.23
Spain	Murcia, Región de	1 492 314	2 566	144	0.96	(0.56, 3.1)	(0.62, 2.27)	1.4	-0.44	-0.31
Spain	Madrid	6 667 630	71 631	8 847	11.79	(6.82, 37.97)	(7.54, 27.8)	11.3	0.49	0.04
Spain	Melilla	84 180	134	2	0.33	(0.19, 1.06)	(0.21, 0.78)	1.8	-1.47	-0.82
Spain	Navarra	650 423	8 293	503	6.2	(3.58, 19.96)	(3.96, 14.62)	5.8	0.4	0.07
Spain	Pais Vasco	2 175 306	18 733	1 459	4.78	(2.76, 15.38)	(3.05, 11.26)	4	0.78	0.19
Spain	La Rioja	313 302	5 417	349	8.28	(4.79, 26.67)	(5.29, 19.52)	3.4	4.88	1.44
Spain	Valenciana, Comunidad	4 988 336	14 680	1 366	2.3	(1.33, 7.4)	(1.47, 5.41)	2.4	-0.1	-0.04
U.S.	New York City	8 609 745	184 197	15 009	19.67	(11.37, 63.41)	(12.57, 46.42)	21	-1.33	-0.06
U.S.	New York	19 428 618	379 482	24 348	11.87	(6.87, 38.25)	(7.59, 28.00)	12.5	-0.63	-0.05
U.S.	Connecticut	3 558 382	29 287	2 436	6.17	(3.57, 19.88)	(3.94, 14.55)	4.94	1.23	0.25
U.S.	Missouri	6 118 556	7 275	374	0.59	(0.34, 1.89)	(0.37, 1.38)	2.65	-2.06	-0.78
Brazil	Porto Velho	541 520	5 161	171	8.95	(5.17, 28.85)	(5.72, 21.11)	3.2	5.75	1.8
Brazil	Cruzeiro Do Sul	84 681	1 359	17	5.58	(3.23, 18)	(3.57, 13.18)	15.5	-9.92	-0.64
Brazil	Rio Branco	409 494	4 545	151	9.66	(5.59, 31.14)	(6.18, 22.8)	4.7	4.96	1.06
Brazil	Lábrea	47 409	117	0	0	(0, 0)	(0, 0)	3.7	-3.7	-1
Brazil	Manaus	2 215 272	20 837	1 466	18.11	(10.47, 58.38)	(11.58, 42.73)	14.6	3.51	0.24
Brazil	Parintins	110 739	1 658	60	14.26	(8.25, 45.96)	(9.12, 33.64)	11.3	2.96	0.26
Brazil	Tefé	56 015	2 253	62	33.14	(19.16, 106.87)	(21.18, 78.22)	20.2	12.94	0.64
Brazil	Boa Vista	405 175	4 423	122	9.84	(5.69, 31.75)	(6.29, 23.24)	25.4	-15.56	-0.61
Brazil	Rorainópolis	33 761	187	4	3.88	(2.24, 12.51)	(2.48, 9.16)	10.3	-6.42	-0.62
Brazil	Altamira	113 860	612	16	3.54	(2.04, 11.4)	(2.26, 8.34)	2.8	0.74	0.26
Brazil	Belém	1 358 828	14 091	1 588	20.15	(11.65, 64.95)	(12.88, 47.55)	16.9	3.25	0.19
Brazil	Breves	95 812	632	63	18.81	(10.87, 60.64)	(12.02, 44.38)	12.2	6.61	0.54
Brazil	Castanhal	200 680	966	117	14.18	(8.2, 45.7)	(9.06, 33.45)	10.8	3.38	0.31
Brazil	Marabá	292 674	797	103	10.79	(6.24, 34.78)	(6.9, 25.46)	10.3	0.49	0.05
Brazil	Redenção	80 586	300	2	0.59	(0.34, 1.91)	(0.38, 1.4)	1.4	-0.81	-0.58
Brazil	Santarém	282 693	1 364	108	8.61	(4.98, 27.76)	(5.51, 20.32)	10.8	-2.19	-0.2
Brazil	Macapá	510 529	5 579	162	9.63	(5.57, 31.06)	(6.16, 22.74)	15	-5.37	-0.36
Brazil	Oiapoque	29 428	562	6	8.7	(5.03, 28.06)	(5.56, 20.54)	5.1	3.6	0.71
Brazil	Araguaína	187 380	2 347	23	2.98	(1.72, 9.59)	(1.9, 7.02)	1.1	1.88	1.71
Brazil	Bacabal	99 418	720	12	2.3	(1.33, 7.41)	(1.47, 5.43)	4.7	-2.4	-0.51
Brazil	Imperatriz	241 852	2 671	122	10.38	(6, 33.46)	(6.64, 24.49)	16.5	-6.12	-0.37
Brazil	Presidente Dutra	46 544	288	1	0.43	(0.25, 1.38)	(0.27, 1.01)	8.4	-7.97	-0.95
Brazil	São Luís	1 079 399	10 613	568	10.84	(6.27, 34.95)	(6.93, 25.59)	6.6	4.24	0.64
Brazil	Parnaíba	153 470	911	15	1.76	(1.02, 5.68)	(1.13, 4.16)	5.6	-3.84	-0.69
Brazil	Teresina	889 475	3 309	137	3.15	(1.82, 10.16)	(2.01, 7.44)	1.4	1.75	1.25
Brazil	Fortaleza	2 649 409	27 411	2 458	16.59	(9.59, 53.47)	(10.6, 39.14)	15.6	0.99	0.06
Brazil	Juazeiro Do Norte	282 369	476	21	1.42	(0.82, 4.56)	(0.9, 3.34)	1.4	0.02	0.01
Brazil	Quixadá	88 861	846	27	5.27	(3.05, 16.99)	(3.37, 12.44)	1.4	3.87	2.77
Brazil	Natal	866 761	4 139	145	2.74	(1.58, 8.82)	(1.75, 6.46)	3.4	-0.66	-0.2
Brazil	Campina Grande	404 576	2 473	44	1.81	(1.05, 5.85)	(1.16, 4.28)	6.6	-4.79	-0.73
Brazil	João Pessoa	846 574	6 004	170	3.51	(2.03, 11.32)	(2.25, 8.29)	6.1	-2.59	-0.42
Brazil	Patos	107 252	754	25	4.03	(2.33, 12.98)	(2.57, 9.5)	1.4	2.63	1.88
Brazil	Caruaru	383 031	554	43	2.24	(1.3, 7.23)	(1.43, 5.29)	2.1	0.14	0.07
Brazil	Recife	1 605 219	16 909	1 242	11.44	(6.62, 36.88)	(7.31, 27)	3.2	8.24	2.58
Brazil	Arapiraca	236 127	847	22	1.85	(1.07, 5.98)	(1.19, 4.38)	2.8	-0.95	-0.34
Brazil	Maceió	1 041 302	8 372	391	7.36	(4.25, 23.72)	(4.7, 17.36)	12.2	-4.84	-0.4
Brazil	Itabaiana	94 735	421	6	1.28	(0.74, 4.12)	(0.82, 3.01)	1.4	-0.12	-0.09
Brazil	Salvador	2 829 205	16 001	600	3.63	(2.1, 11.7)	(2.32, 8.56)	5.4	-1.77	-0.33
Brazil	Cachoeiro De Itapemirim	207 783	573	18	1.3	(0.75, 4.19)	(0.83, 3.07)	1.4	-0.1	-0.07
Brazil	Vitória	370 821	3 508	126	4.65	(2.69, 14.99)	(2.97, 10.97)	3.2	1.45	0.45
Brazil	Rio De Janeiro	6 580 483	36 115	4 462	8.3	(4.8, 26.73)	(5.3, 19.57)	7.5	0.8	0.11
Brazil	São Paulo	12 126 244	74 796	4 920	5.86	(3.39, 18.87)	(3.74, 13.81)	2.3	3.56	1.55
Brazil	Cuiabá	578 557	1 212	24	0.79	(0.46, 2.55)	(0.51, 1.87)	1.4	-0.61	-0.43
Brazil	Luziânia	208 281	101	4	0.49	(0.28, 1.58)	(0.31, 1.16)	1.4	-0.91	-0.65

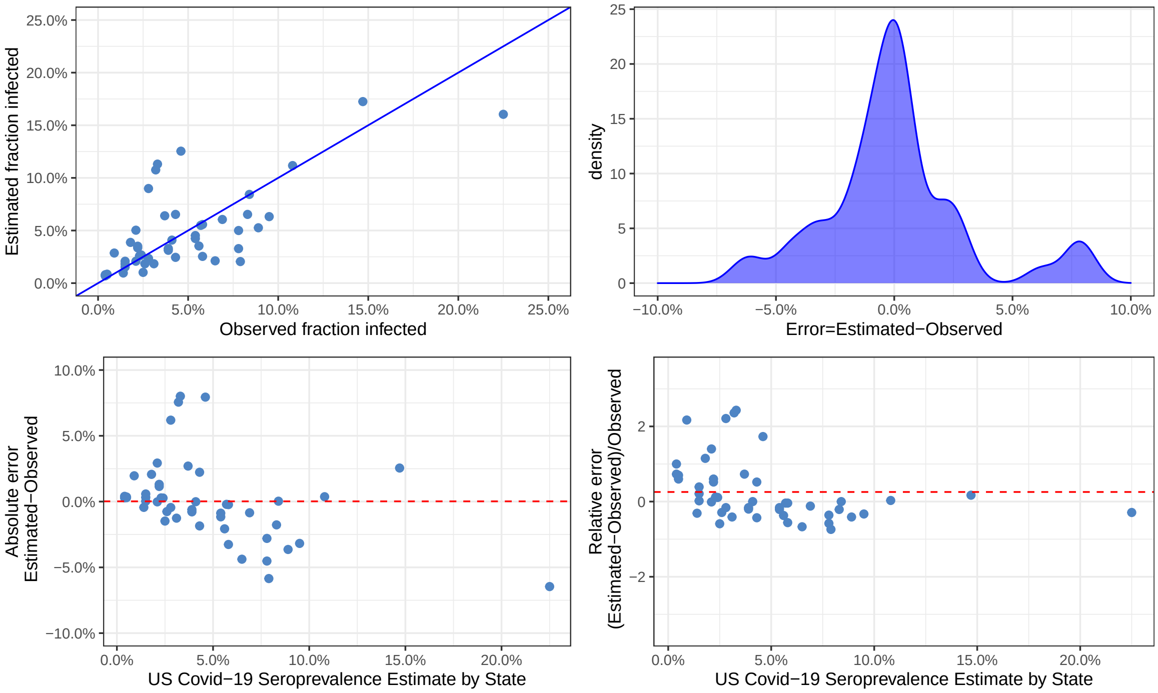

Figure S7: Absolute and relative errors between the estimated fraction of people ever infected and the U.S. Covid-19 seroprevalence estimate by state. *Source:* First round of the "Nationwide Commercial Laboratory Seroprevalence Survey" (CDC) in August. *Notes*: Dashed red lines represent the mean error. The epidemiological data used corresponds to August 28 2020, which is close to 18 days after the U.S. Covid-19 seroprevalence data was taken. The states with the highest absolute error are Connecticut, District of Columbia, Massachusetts, New York, and Rhode Island for which the "Nationwide Commercial Laboratory Seroprevalence Survey" reports prevalence values of 3.3%, 3.2%, 4.6%, 22.5%, and 2.8%, respectively. However, in Connecticut, for instance, the "Ten-Site Survey" from CDC reports a prevalence value of 6.7% by the end of July with a larger sample size [11], which already is more than double the number reported by the "Nationwide Commercial Laboratory Seroprevalence Survey". In the case of New York, round 4 of the "Nationwide Commercial Laboratory Seroprevalence Survey" reports a prevalence value of 17%, which is closer to our estimated value.

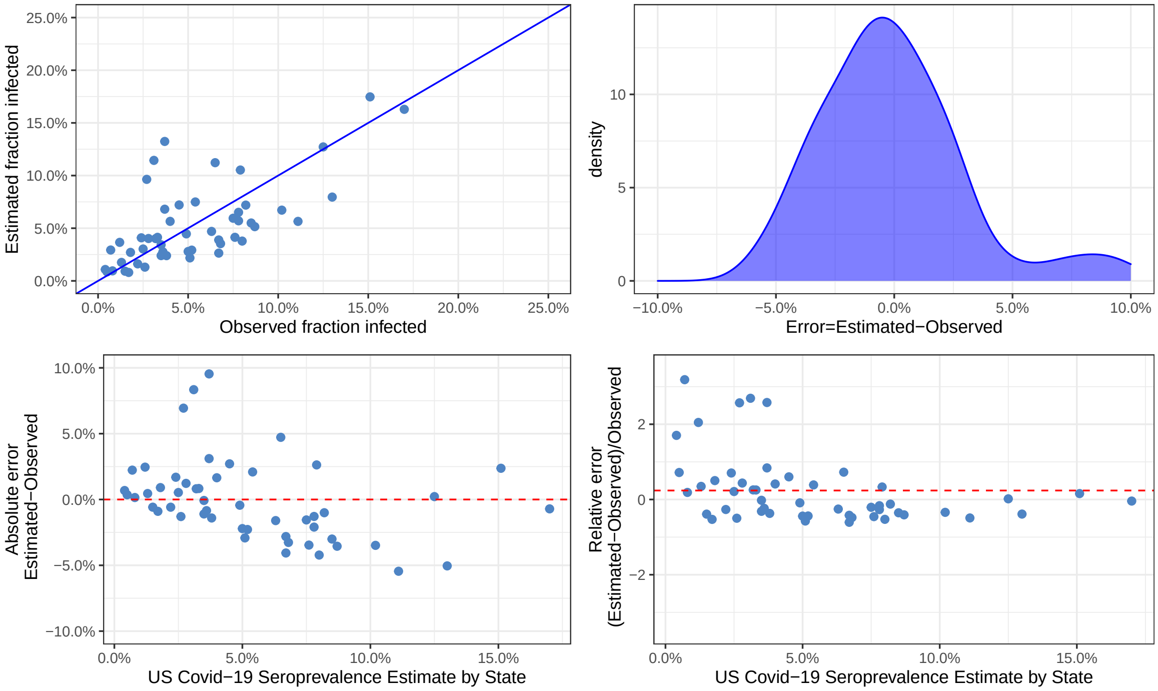

Figure S8: Absolute and relative errors between the estimated fraction of people ever infected and the U.S. Covid-19 seroprevalence estimate by state. *Source:* Fourth round of the "Nationwide Commercial Laboratory Seroprevalence Survey" (CDC) in September. *Notes*: Dashed red lines represent the mean error. The epidemiological data used corresponds to October 5 2020, which is close to 18 days after the U.S. Covid-19 seroprevalence data was taken. The states with the highest absolute error are Connecticut, Massachusetts, and Rhode Island for which the "Nationwide Commercial Laboratory Seroprevalence Survey" reports prevalence values of 3.1%, 3.7%, and 2.7%, respectively. However, in Connecticut, for instance, the "Ten-Site Survey" from CDC reports a prevalence value of 6.7% by the end of July with a larger sample size [11], which already is more than double the number reported by the "Nationwide Commercial Laboratory Seroprevalence Survey".
